# Euryhaline fish larvae ingest more microplastic particles in seawater than in freshwater

**DOI:** 10.1038/s41598-023-30339-y

**Published:** 2023-03-10

**Authors:** Hilda Mardiana Pratiwi, Toshiyuki Takagi, Suhaila Rusni, Koji Inoue

**Affiliations:** 1 Graduate School of Frontier Sciences, The University of Tokyo, Kashiwa, 277-8563 Japan; 2 Atmosphere and Ocean Research Institute, The University of Tokyo, Kashiwa, 277-8564 Japan

**Keywords:** Physiology, Environmental sciences, Ocean sciences

## Abstract

Microplastic (MP) pollution is a major concern in aquatic environments. Many studies have detected MPs in fishes; however, little is known about differences of microplastic uptake by fish in freshwater (FW) and those in seawater (SW), although physiological conditions of fish differ significantly in the two media. In this study, we exposed larvae (21 days post-hatching) of *Oryzias javanicus* (euryhaline SW) and *Oryzias latipes* (euryhaline FW), to 1-µm polystyrene microspheres in SW and FW for 1, 3, or 7 days, after which, microscopic observation was conducted. MPs were detected in the gastrointestinal tracts in both FW and SW groups, and MP numbers were higher in the SW group in both species. Vertical distribution of MPs in the water, and body sizes of both species exhibited no significant difference between SW and FW. Detection of water containing a fluorescent dye revealed that *O. javanicus* larvae swallowed more water in SW than in FW, as has also been reported for *O. latipes*. Therefore, MPs are thought to be ingested with water for osmoregulation. These results imply that SW fish ingest more MPs than FW fish when exposed to the same concentration of MPs.

## Introduction

Microplastics (MPs) are generally defined as plastic particles smaller than 5 mm^1^. In recent decades, MPs have become a major global issue in freshwater (FW) and marine environments^2,3^. MPs can have adverse effects on aquatic organisms, including fish. Recent studies have detected MPs in the bodies of both FW and marine fish^4–8^, mostly in the gastrointestinal tracts and gills of fish^9–11^.

Marine and FW fish experience very different physiological conditions, since osmotic pressure in the two media differs greatly. As marine fish are constantly dehydrated by hyperosmotic seawater (SW), they drink ambient SW almost continuously to compensate for the water loss. From ingested SW, marine fish adsorb sodium and chloride ions in the intestine, and then absorb water, the osmolality of which is reduced by this desalination. Absorbed monovalent ions are eliminates by chloride cells in the gill. In contrast, hypoosmotic FW tends to diffuse into FW fish, due to the osmotic pressure difference; thus, FW fish drink less water^12,13^. Such differences in water uptake and osmoregulation may influence MP ingestion. However, previous studies of MPs in fish have investigated either FW or marine species only^14–16^, thus the difference of MP ingestion in the two media was not clear because no reports have compared MP uptake in FW and SW using the same species.

Here, we employed medaka (*Oryzias*) fishes to demonstrate differences in MP uptake in the two different media. Medaka are an attractive model fish for ecotoxicology studies because of their low rearing cost, high fertility, ease in rearing of embryos, abundant developmental information, and availability of a complete genome sequence^17–21^. Moreover, a major advantage of medaka is the availability of related species that inhabit different osmotic environments with different euryhalinities^22,23^. In this study, we used two species, Japanese medaka (*Oryzias latipes*), which inhabits FW, but which adapts to SW, and Javanese medaka (*Oryzias javanicus*), which inhabits SW/brackish water, but which adapts to FW^22,23^. We used larvae (third larval stage; stage 42, 21 days post-hatching [dph])^19^ as a model animal. Larvae at this developmental stage have internal organs already developed as in adult fish, despite their relatively small body size, which is beneficial for easier in situ observation of ingested MPs. Here, we exposed medaka larvae reared in different media (SW and FW) to fluorescent-labeled polystyrene particles (1 µm in size) for 7 days and detected these particles in their bodies using a fluorescence microscope. We suggest that the difference in MP uptake in SW and FW environments is caused by the difference in water consumption.

## Results

### Microplastic distribution in the larval body

**Figure 1:**
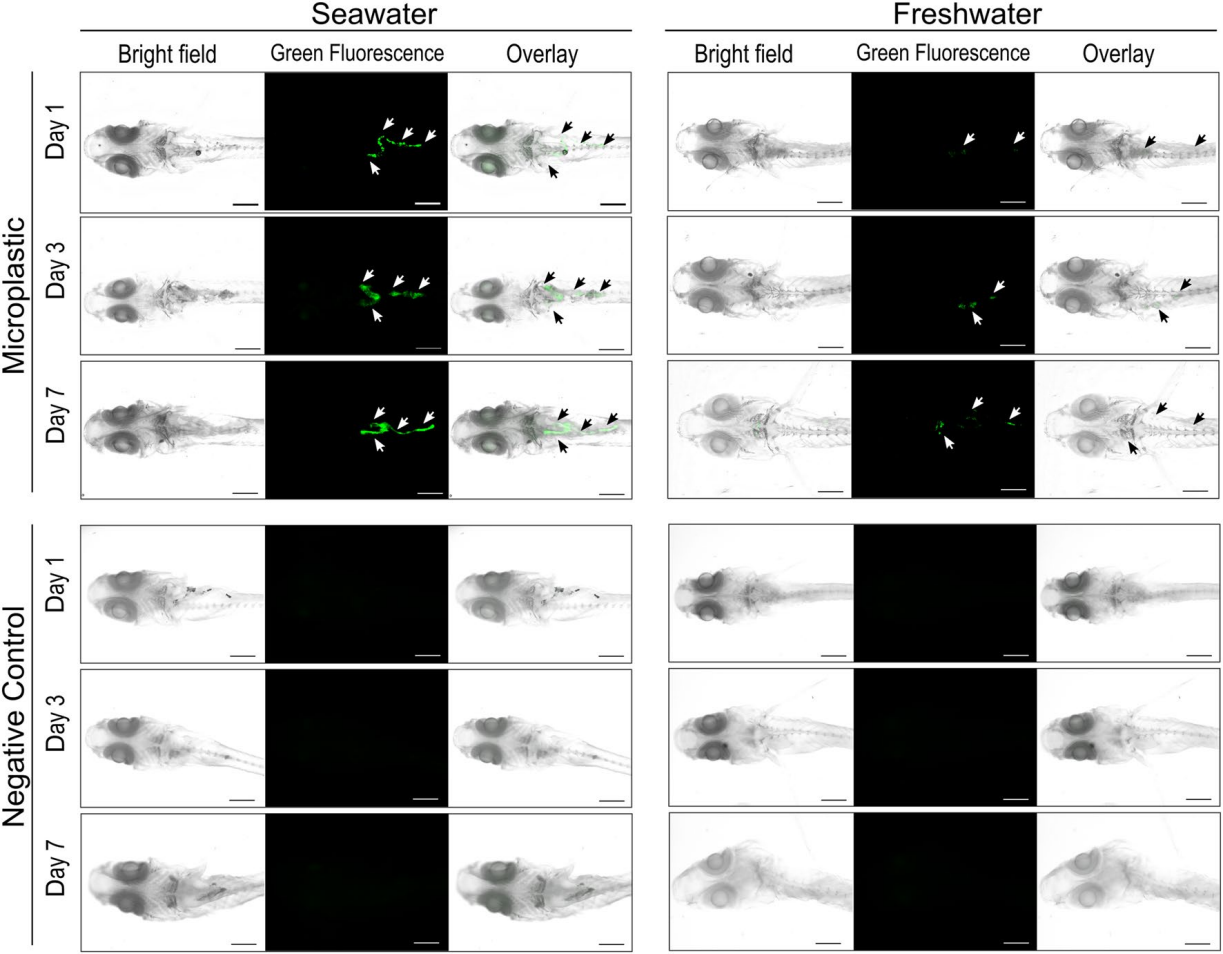
Microplastic distribution in the gastrointestinal tract of *Oryzias javanicus* (*Oj*) reared in seawater and freshwater. Images show *Oj* larvae exposed to fluorescently labeled microplastics at 0.25 mg/L for 1, 3, or 7 days. Images for negative controls were taken of *Oj* larvae cultured in water without microplastics for 1, 3, or 7 days. The scale bar indicates 0.5 mm. White and black arrowheads show the green fluorescent signal from microplastics in the gastrointestinal tracts of larvae. Caudal fins are not shown in this figure.

**Figure 2:**
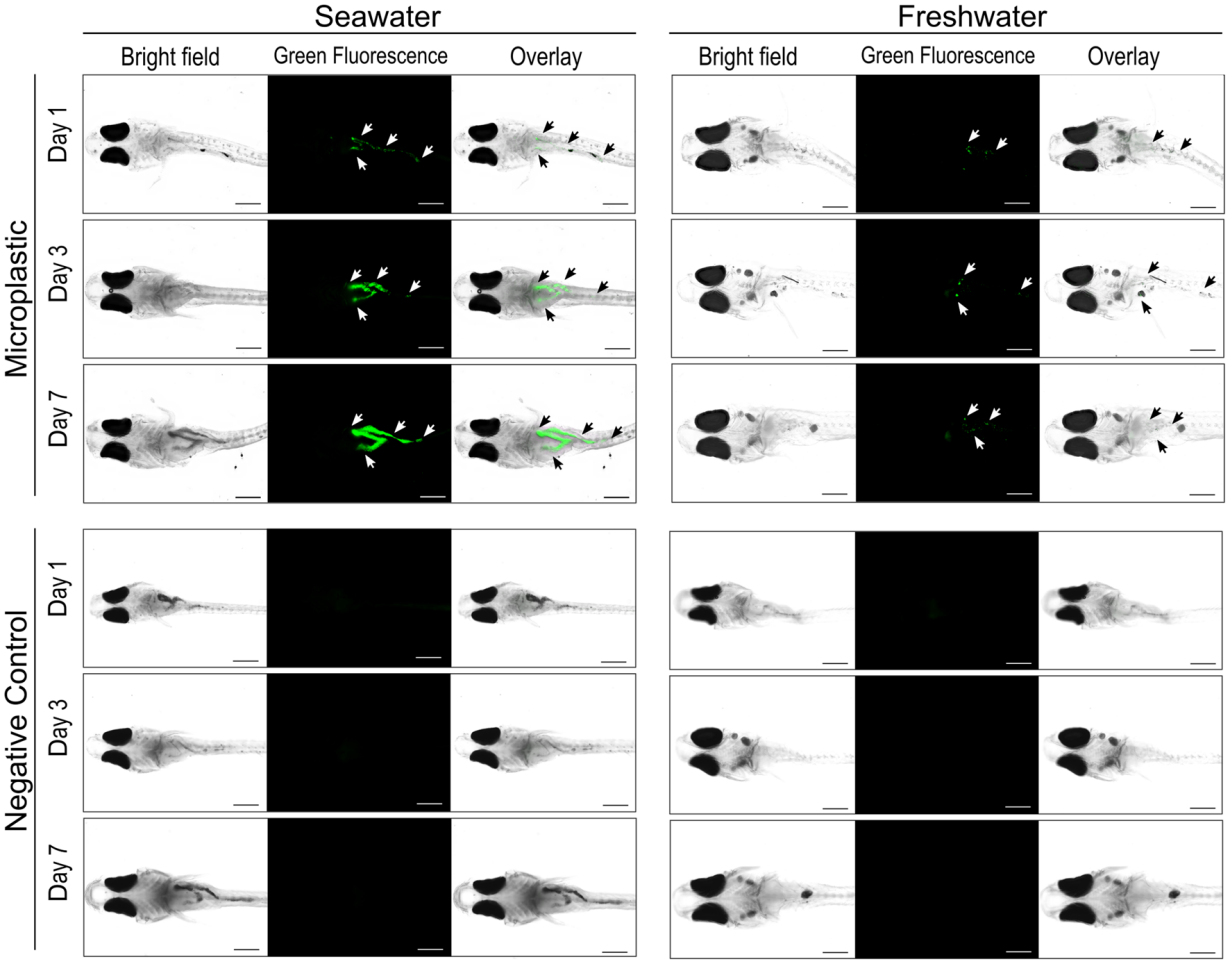
Microplastic distribution in the gastrointestinal tracts of *Oryzias latipes* (*Ol*) reared in seawater and freshwater. Images show *Ol* larvae exposed to fluorescently labeled microplastics at 0.25 mg/L for 1, 3, or 7 days. Images for negative controls were taken of *Ol* larvae cultured in water without microplastics for 1, 3, or 7 days. The scale bar indicates 0.5 mm. White and black arrowheads show the green fluorescent signal from microplastic in the gastrointestinal tracts of larvae. Caudal fins are not shown in this figure.

We exposed *O. javanicus* larvae (21 dph) that had been reared in 31-parts per thousand (ppt) SW (SW-*Oj*) and larvae acclimated to FW (FW-*Oj*) to 1-µm MPs for up to 7 days. After 1, 3, or 7 days of exposure, larvae were fixed and transparentized, and MP distribution was observed under a fluorescence microscope. We found that most MPs accumulated in the guts of both SW-*Oj* and FW-*Oj*, and that SW-*Oj* showed higher accumulations than FW-*Oj* (Fig. 1). We also observed some MPs near eye vesicles, but their positions were not determined precisely because tissues became less well-defined during transparentization (Supplementary Fig. S1). A similar experiment was conducted using *O. latipes* larvae reared in FW (FW-*Ol*) and those acclimated to 20-ppt SW (SW-*Ol*). We used SW-*Ol* acclimated to 20-ppt SW because exposure to 31-ppt SW caused high mortality in a preliminary experiment (Supplementary Fig. S2). Results were almost the same as with *O. javanicus*. MPs accumulated in the gut and were more abundant in SW-*Ol* than FW-*Ol* (Fig. 2). Fluorescent signals were not observed in transparentized negative control larvae of either species (Figs. 1, 2).

### Comparison of microplastic numbers accumulated in larvae

**Figure 3:**
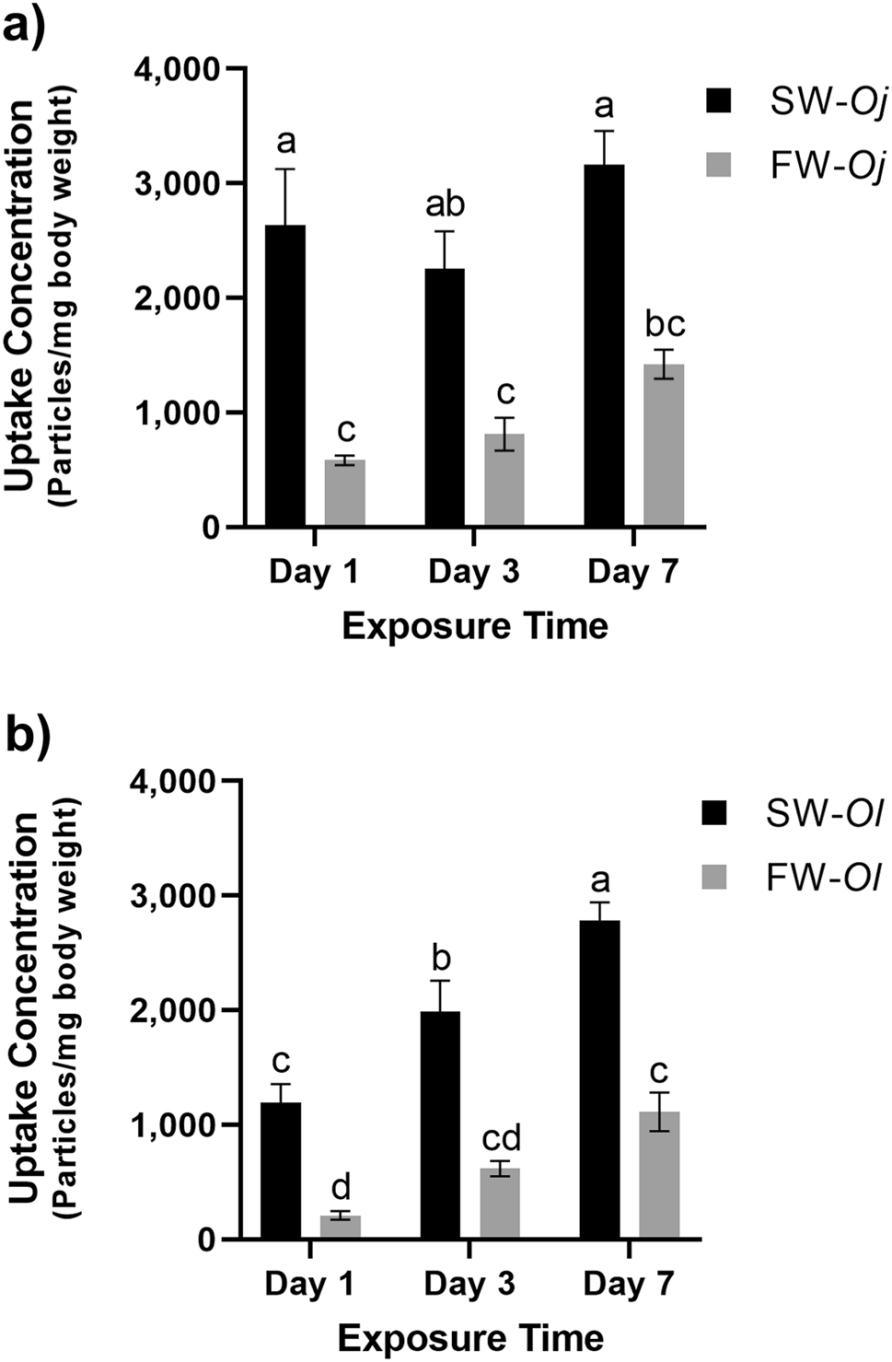
Microplastic uptake in medaka larvae. (**a**) *Oryzias javanicus* (*Oj*), (**b**) *O. latipes* (*Ol*). Larvae were exposed to microplastics (0.25 mg/L) for 1, 3, or 7 days. The black bar (SW-*Oj*, SW-*Ol*) indicates uptake concentrations in seawater groups, whereas the grey bar (FW-*Oj*, FW-*Ol*) shows uptake concentrations in freshwater groups. Each bar represents the mean of uptake concentration ± standard error of the mean (SEM). Different letters above the bars indicate significant differences, which were analyzed using one-way analysis of variance (ANOVA) (P < 0.05) followed by Tukey’s Honestly Significant Difference post-hoc test.

MPs in larvae were extracted, filtered, and counted using a fluorescence microscope. Results were normalized by body weight (Fig. 3). No MPs were detected in negative controls in any exposure group, showing that no MP contamination occurred. In *O. javanicus*, MP numbers were higher in seawater exposure groups (SW-*Oj*) than freshwater groups (FW-*Oj*) throughout the experimental period (Fig. 3a). Similar results were also found in *O. latipes*. MP numbers in SW-*Ol* were higher than in FW-*Ol* fish (Fig. 3b). Differences in mean MP numbers between SW and FW fish in both species were statistically significant (P < 0.05) in all sampling periods. These results suggest that fish in SW ingest greater number of MPs when exposed to the same MP concentrations. MP uptake showed a gradual increase with exposure time (day 1, 3, and 7) in all exposure groups, and differences were statistically significant on days 1, 3, 7 for SW-*Ol*, and between days 1 and 7 for FW-*Ol* (Fig. 3b).

### Vertical distribution of microplastics in seawater and freshwater

**Figure 4:**
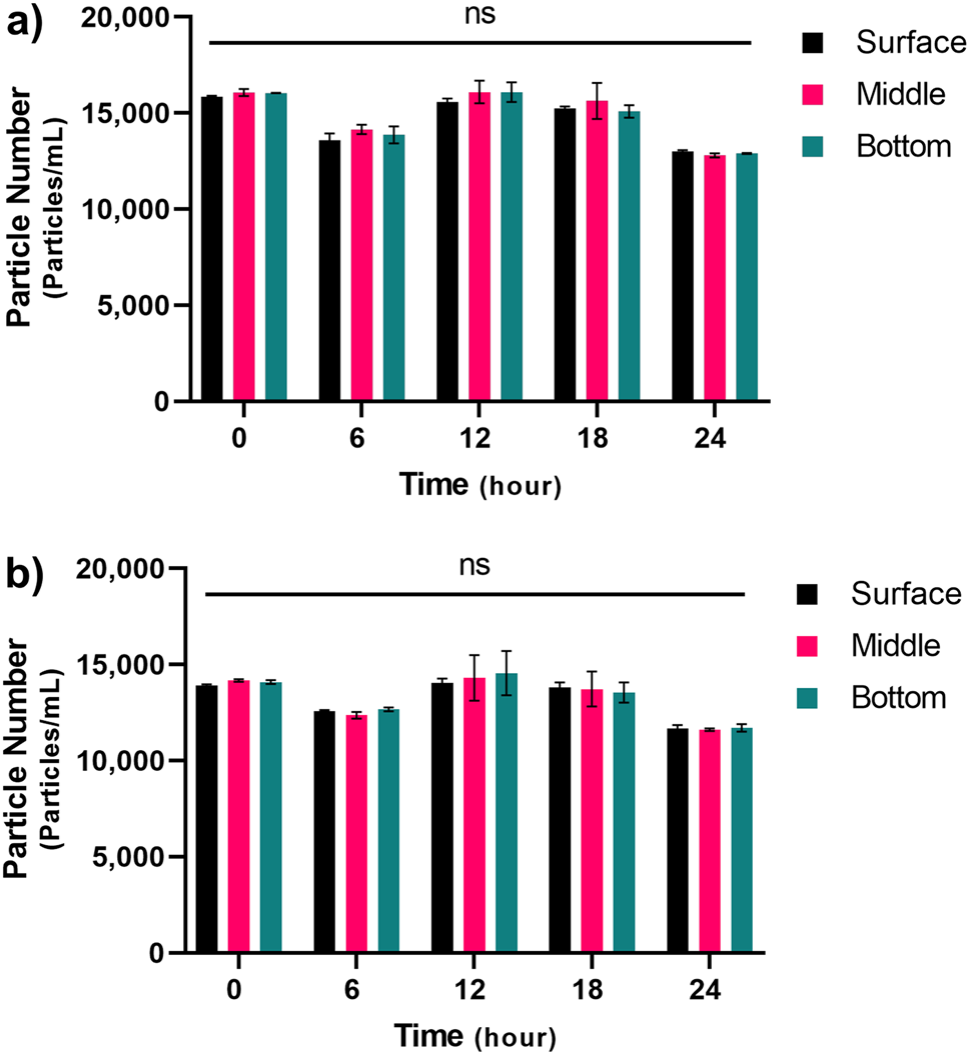
Microplastic vertical distribution. (**a**) Seawater, (**b**) Freshwater. Vertical distributions of polystyrene microplastics (0.25 mg/L) at the surface, middle, and bottom of the solution were measured using a microplate reader. The black bar indicates the MP particle number at the surface. The magenta bar indicates the MP particle number in the middle, whereas the blue bar shows the concentration at the bottom of the solution in seawater and freshwater. Differences between areas were analyzed for significance using one-way ANOVA (*ns* non-significant P > 0.05).

We considered the possibility that a difference in MP buoyancy in the two media might have contributed to the difference in MP uptake; therefore, MP vertical distribution was examined in FW and SW using the same beakers and aeration system as in the exposure experiment. This comparison revealed that MPs were distributed evenly from the surface to the bottom in SW (P = 0.4607) and FW (P = 0.2001) (Fig. 4). Accordingly, the difference in MP uptake in SW and FW cannot be attributed to MP buoyancy differences.

### Comparison of morphological features between seawater and freshwater larvae

**Figure 5:**
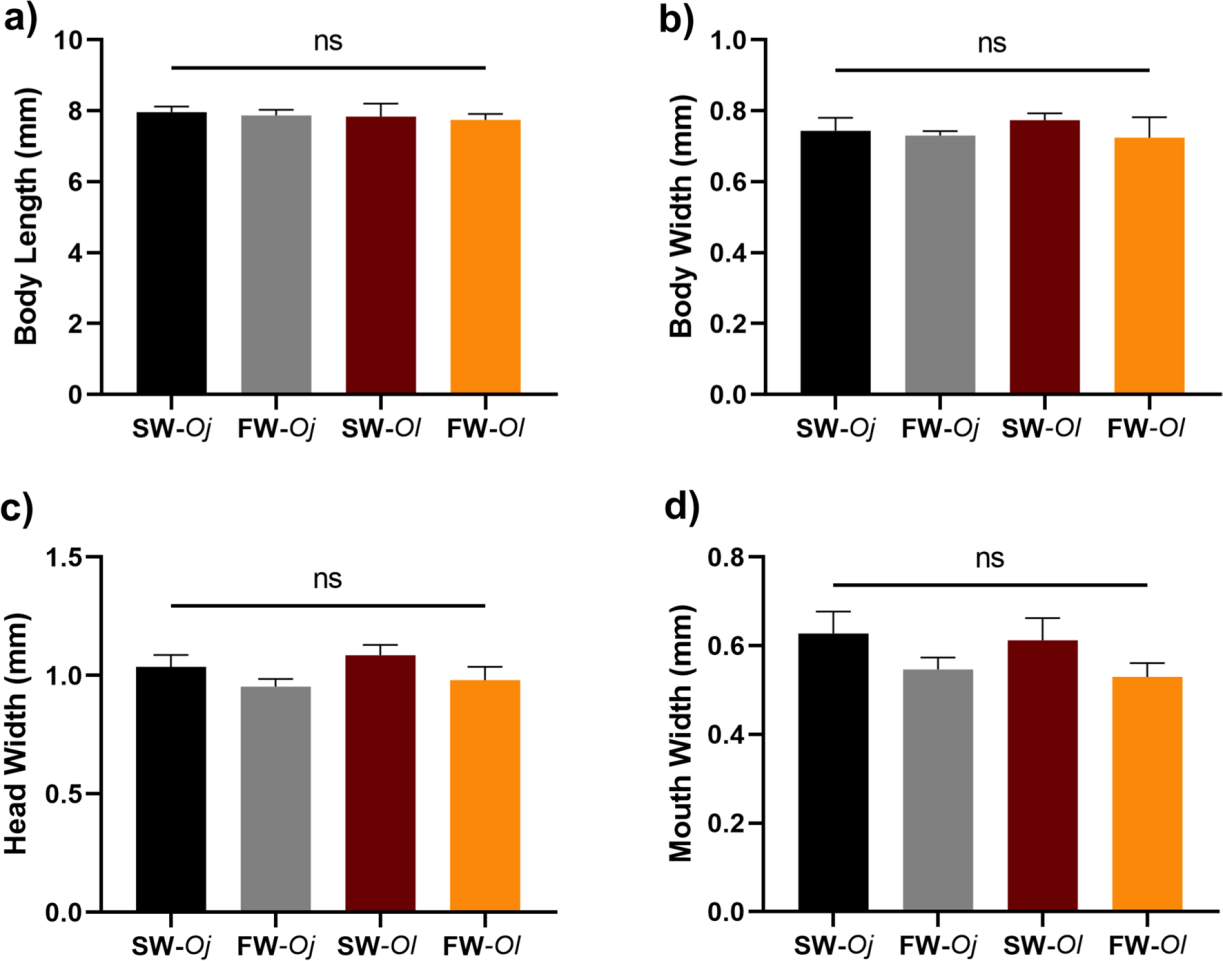
Body Measurements of *Oryzias javanicus* and *O. latipes* larvae reared in freshwater and seawater. (**a**) Body length, (**b**) Body width, (**c**) Head width, (**d**) Mouth width. Morphological features of each medaka group were measured using a microscope. The black bar indicates seawater-reared *O. javanicus* (SW-*Oj*). The grey bar indicates freshwater-reared *O. javanicus* (FW-*Oj*). The brown bar shows seawater-reared *O. latipes* (SW-*Ol*), whereas the orange bar shows freshwater-reared *O. latipes* (FW-*Ol*). Differences between areas were analyzed for significance using one-way ANOVA (*ns* non-significant P > 0.05).

We also considered the possibility that larval body sizes may have differed between FW and SW, and that such a size difference might have caused the difference of MP uptake. Thus, total length, mouth width, head width, and body width were measured and compared among SW-*Oj*, FW-*Oj*, SW-*Ol* and FW-*Ol* larvae at 21 days post-hatching (Fig. 5). Nonetheless, no statistically significant differences in any measurements were found among groups. However, SW larval groups (SW-*Oj* and SW-*Ol*) showed slightly greater values in mouth and head width measurements compared to FW groups (FW-*Oj* and FW-*Ol*) (Fig. 5c,d).

### Water drinking observation

Water drinking in larvae of SW-*Oj* and FW-*Oj* was compared using SW and FW containing fluorescein isothiocyanate (FITC)-labeled dextran. After 3 h immersion in the respective media, FITC signals were observed under a fluorescence microscope. FITC signals were found in all SW-*Oj* larvae, except FW-*Oj* larvae (n = 3) (Supplementary Fig. S3).

## Discussion

In this study, we compared MP uptake under different salinities using two species of medaka fishes, *O. javanicus* and *O. latipes*. *O. javanicus* larvae in SW (SW-*Oj*) ingested more MPs than those in FW (FW-*Oj*) (Figs. 1, 3a). Although *O. javanicus* can adapt to FW^22,23^, its native habitat is SW or brackish water (BW)^24^. Considering that *O. javanicus* is under stress in FW, the same experiment was conducted using *O. latipes*, which is a FW fish that can adapt to SW. When exposed to MPs, larvae adapted to 20-ppt SW accumulated more MPs than those reared in FW (Figs. 2, 3b). We used *O. latipes* larvae acclimatized to 20-ppt SW because exposure to concentrations higher than 20-ppt caused high mortality (Supplementary Fig. S2). Although it has been reported that adult *O. latipes* can adapt to 35-ppt SW^22^, larvae 7 to 21 dph may be less adaptable to SW^25^. Salinity of 20-ppt is unquestionably hyperosmotic for *O. latipes* larvae^26^. Thus, we concluded that larvae of both medaka fishes ingest more MPs in SW than FW, irrespective of whether the native habitat is SW, BW, or FW.

As SW and FW have different densities (Supplementary Table S1), we assumed that the vertical distribution of MPs might differ in the two media. Since medaka prefer the water surface^24,27^, the vertical distribution of MPs might have caused a difference in uptake. Therefore, we compared the vertical distribution of MPs in FW and SW under our experimental conditions. However, we found that MPs were uniformly distributed from the surface to the bottom in both SW and FW, suggesting that vertical MP distribution was not a factor.

We also compared total length, body width, head width, and mouth width of SW- and FW-acclimated larvae of both species, considering the possibility that osmotic stress affected body size, because growth hormone is involved in seawater adaptation in some fishes^28^. However, none of these parameters differed significantly, indicating that differences of MP uptake were not caused by differences of body size. Interestingly, mouth and head widths of seawater larvae (SW-*Oj* and SW-*Ol*) tended to be greater compared than those of the freshwater medaka (FW-*Oj* and FW-*Ol*) (Fig. 5), although these differences were not statistically significant. Implications of this result will be discussed later.

Previous studies have proposed several pathways of MP uptake in fish, such as by ingestion due to food/prey misidentification, transfer via the food chain, and passive ingestion via water drinking^29^. In all four experimental groups, MP particles were detected predominantly in the gut. This suggests that particles were ingested. The 1-µm MP particles used in this study were too small to be mistaken for food; therefore, we think that the main factor affecting MP uptake in *O. javanicus* and *O. latipes* larvae is water drinking for osmoregulation. Basically, osmolality of fish body fluids is maintained at approximately one third that of SW, in both SW fish and FW fish; thus, SW is hyperosmotic and FW is hypoosmotic for teleosts. Accordingly, teleosts must regulate body fluid osmolality in opposite directions in SW and FW, and euryhaline fishes can conduct both FW- and SW-type osmoregulation^30^. Teleosts in SW drink water to compensate for water loss in high salinity environments. They desalinize ingested SW in the esophagus and intestine, and then absorb water^12^. Thus, drinking is critical for adaptation to hyperosmotic environments. In contrast, FW fishes drink less water because a large amount of water enters their tissues by osmosis^31^. In *O. latipes* larvae, more water drinking in SW than FW has been reported^32^. In the present study, we compared water drinking in SW- and FW-acclimated *O. javanicus* and demonstrated higher water uptake in SW than in FW (Supplementary Fig. S3). Moreover, in this study, the mass of MPs in the guts of SW-*Oj* was approximately 4.5 times higher than of FW-*Oj*, and that of SW-*Ol* was 6 times higher than FW-*Ol*. Higher water drinking rates in SW than in FW have been reported in many other euryhaline teleosts^13,33–36^. Therefore, increased MP uptake in SW is likely to occur generally in euryhaline teleosts, and marine teleosts may ingest more MPs than FW teleosts, given the same concentration of MPs in the environment.

The tendency for wider bodies and mouths in SW-reared larvae, mentioned above, may be related to active drinking rates. Larvae in SW are presumed to open their mouths more frequently, to drink water, than those in FW. Bone staining of teleost larvae in previous reports show that the heads and mouths of teleost larvae initially consist of cartilaginous tissues, and ossification of the head and mouth commences later^37,38^. Medaka larvae used in this study correspond to stages with cartilaginous heads and mouths. Since cartilage is soft, elastic, flexible skeletal tissue, we assumed that enhanced drinking in early developmental stages may influence head and mouth structure. This idea is consistent with previous articles, reporting that physical activity can affect cartilage development in healthy human children^39^.

The concentration of MPs ingested in this study showed a gradual increase with exposure time in all exposure groups, especially in *O. latipes* (Fig. 3). This result was consistent with a previous report, using seabass, showing increased organ MP accumulation with longer exposure^40^. These results imply that the rate of elimination of MP particles is slower than the rate of ingestion, and that fish in both FW and SW accumulate MP particles, although rates of accumulation may differ, depending on particle size, shape, and concentration^41^.

Recent studies using different species of freshwater and seawater fish also reported that MP gut retention time varies by species^42,43^. Therefore, differences in uptake of MP particles cannot be inferred by simple comparisons of different species of marine and FW fishes. In this study, using two euryhaline *Oryzias* species, we clearly demonstrated that fish in SW ingest more MP particles than those in FW.

Most MP pollution originates on land as a result of anthropogenic activity, and MPs are carried by rivers to the sea^44^. The mean MP concentration in 14 Asian countries ranged from 100 items m^−3^ to more than 5000 items m^−3^ in FW environments^45^. The mean MP concentration was estimated as 0.44 items m^−3^ globally in marine environment^46^, much lower than the concentration in FW. Additionally, MP masses range from 0.4–250 μg/L in SW^47^ and 30–1790 μg/L in FW^48^. Accordingly, the concentration of 1-µm PS-MPs used in this study was within environmentally relevant levels. We suggest that MP uptake is higher in marine ecosystems than in freshwater environments. Therefore, even though concentrations of MPs in freshwater are higher than in seawater, the risk to marine ecosystems should not be dismissed, and marine regions with high MP concentrations should be monitored carefully.

## Materials and methods

### Experimental organisms

Javanese medaka (*Oryzias javanicus*; *Oj*) strain (RS831) from Penang, Malaysia, were obtained from the National Bioresource Project (NBRP) MEDAKA at the National Institute for Basic Biology, Okazaki, Japan. These fish were maintained in an aquarium system (Iwaki Co., Tokyo, Japan) with recirculating seawater. Seawater salinity was maintained at ~ 31-ppt. Additionally, an orange-red variant of *O. latipes* (*Oryzias latipes*; *Ol*) was purchased from a commercial source (Charm Co., Ltd., Gunma, Japan). *O. latipes* were cultured in freshwater using a 30-L tank equipped with standard aquarium filters and an air pump. Both species were fed twice a day with brine shrimp (*Artemia nauplii*) (INVE Aquaculture, Belgium) larvae. The photoperiod was set to 14/10 h day/night at 26 °C. Under such conditions, adult fish of both species spawn every morning.

All animal experiments complied with the ARRIVE guidelines (Animal Research: Reporting of In Vivo Experiments). Care and use of animals were conducted according to Guidelines for the Care and Use of Animals from The University of Tokyo. All experimental procedures were approved by the Animal Ethics Committee of the Atmosphere and Ocean Research Institute of the University of Tokyo (permission no. P18-13).

### Microplastic particles

Polystyrene MPs (PS-MPs; Diameter 1 µm with a coefficient of variation 2%) labeled with a fluorescent dye (yellow green; excitation 441.53 nm and emission 485.56 nm) were purchased from Polyscience, Inc., Warrington, PA, USA (Catalog No. 17154-10). PS microspheres (Density: 1.05 g/cm^3^) were provided as a suspension in water and were easily dispersed without aggregation. The PS-MP stock solution was stored at 4 °C.

### Larval acclimation at different salinities

All acclimation processes were conducted at 26 °C. Fertilized eggs of *O. javanicus*, obtained by mass mating in 31-ppt SW, were incubated until hatching in petri dishes in 31-ppt SW. To acclimate larvae to FW, half the hatched larvae were maintained in 31-ppt for 7 days, and then acclimated to 15-ppt SW for 7 days, and to FW for 7 days (FW-*Oj*). Another half of *O. javanicus* larvae were maintained in 31-ppt SW for 21 days (SW-*Oj*). Fertilized eggs of *O. latipes*, obtained by mass mating in FW, were incubated until hatching in petri dishes in FW. To acclimate them to 20-ppt SW, half the hatched larvae were maintained in FW for 7 days, and subsequently acclimated to 10-ppt SW for 3 days, 12-ppt SW for 3 days, 15-ppt SW for 3 days, and were finally transferred to 20-ppt SW and maintained for 5 days (SW-*Ol*). The other half of *O. latipes* larvae were maintained in FW for 21 days (FW-*Ol*). As preliminary test, *Ol* larvae were also maintained in FW for 7 days, and then acclimated to 15-ppt SW for 7 days, and to 31-ppt SW for 7 days. Surviving and dead larvae were counted every day during the acclimation period.

### Microplastic exposure

Freshwater and seawater used during tests were filtered before experiments using a 0.22 µm Millipore sterile vacuum filtration system (Sigma-Aldrich, Germany) to avoid particle contamination. Larval groups (21 days post-hatching; 7–8 mm in body length) were moved to 500-mL glass beakers equipped with aeration systems to keep MPs distributed evenly. During exposure tests, medaka larvae (n = 36) were exposed to 0.25 mg/L (10^8^ particles/L) of 1-μm PS-MPs for 7-days. In addition, negative controls consisted of larvae (n = 12) in containers without MPs. Larvae were fed with fish larva powder food (Itosui, Tokyo, Japan) once a day. Also, water for negative control and exposure solutions were changed every day to maintain the salinity and MP concentrations during exposure tests. Moreover, water temperature and salinity were recorded before and during exposure tests (Supplementary Table S1). Densities of FW and SW used in this study were derived from temperature and salinity data^49,50^ (Supplementary Table S1). Larvae were randomly sampled (exposure group n = 12; negative control n = 4 in total) from each glass beaker at experimental days 1, 3, and 7. All exposure tests were conducted in triplicate.

### Tissue transparentization and reagents

Fish larvae were bleached and transparentized according to previous reports^51,52^, with several modifications. Formalin (10% formalin solution), potassium hydroxide (KOH), hydrogen peroxide, Triton-X, phosphate-buffered saline (PBS), HEPES, and glycerin were purchased from Wako Pure Chemical Industries Ltd. (Tokyo, Japan). All other chemicals used in this study were of analytical grade. *O. javanicus* larvae (exposure group n = 6; negative control n = 2) were fixed in 10% formalin at 4 °C for 3 days. After removal of formalin, larvae were incubated in pre-bleaching solution (0.3% hydrogen peroxide in PBS) at room temperature overnight. Pre-bleaching solution was replaced with bleaching solution (3% hydrogen peroxide in PBS) and incubated at room temperature overnight. After bleaching, the bleaching solution was replaced with a tissue transparency solution (5% formalin, 5% Triton X-100, 1% KOH in PBS) and incubated at 42 °C for 24 h. In *O. latipes*, which has a less pigmented peritoneum than *O. javanicus*, bleaching was omitted, i.e., the tissue transparency solution was directly added after fixation, and incubated at 42 °C for 48 h. Transparency of each sample was confirmed under the microscope.

### Microplastic extraction and counting

Fish (exposure group n = 6; negative control n = 2) were sacrificed on ice. Larvae were homogenized in 20 mM HEPES buffer solution (pH 7.0) using homogenizing pestles and then centrifuged at 10,000×*g*, 4 °C for 20 min to separate pellets and supernatants. Supernatants were discarded and 10% KOH was added to pellets, which were then reacted at 42 °C for 3 days to degrade tissues. To extract 1-μm PS-MPs, digested tissue solutions were then filtered using 0.22 µm Isopore™ polycarbonate membrane filters with vacuum filtration. Membrane filters were dried overnight and then set on glass slides with cover slips for particle counting.

### Microplastic detection and counting

Detection and counting of MPs were conducted using an all-in-one fluorescence microscope (BZ-X800, KEYENCE, Osaka, Japan) equipped with a GFP filter (excitation 470/40 nm, emission 525/50 nm, dichroic 495 nm). MP distributions in transparentized larvae were observed at × 4 magnification using glass-based dishes. Glycerin was added on the sample to get clearer image and to prevent the sample from drying during observation. To count MPs extracted from homogenized bodies, images of MPs on membranes were automatically captured by the microscope (× 20 magnification), and the number of MPs was analyzed with the automatic counting system of the BZ-X800 Analyzer Software (KEYENCE), based on fluorescence intensity, shape, and size. MP counts were then normalized by the total mass of fish and compared between exposure groups.

### Microplastic vertical distribution in seawater and freshwater

To estimate the cause of the difference in uptake, 0.25 mg/L (10^8^ particles/L) of 1-μm PS-MPs were added to glass beakers of the same size as in the exposure experiment and filled with filtered 31-ppt SW or FW. These beakers were equipped with the same aeration system as the exposure experiment and covered with aluminum foil to avoid fluorescence signal loss due to light. Vertical MP distributions in SW and FW were examined by sampling water from the surface, middle, and bottom parts of the solution at 0, 6, 12, and 24 h. Fluorescence intensity of PS-MPs in the solutions were measured using a Microplate Reader Spectra iD3 (Molecular Devices, LLC, San Jose, CA, USA) at excitation and emission wavelengths of 441 nm and 486 nm. MP particle numbers were estimated by calibrating fluorescence intensities to a standard curve obtained with serial dilutions of fluorescent PS-MP suspensions.

### Larval body measurements

To explore any differences in morphological features possibly related to MP uptake, head and body sizes of larvae in SW-*Oj*, FW-*Ol*, and FW-*Oj* groups were measured. Larvae of SW-*Oj*, FW-*Ol*, and FW-*Oj* (n = 10) were fixed in 10% formalin for 3 days. Morphological measurements of larval heads and bodies were conducted using the KEYENCE microscope and analyzer software. Body lengths (BL), body widths (BW), mouth widths (MW), and head widths (HW) were measured from the dorsal orientation. Body length was measured from the mouth tip to the caudal fin. Body width was measured as the width between the pectoral fins. Mouth width was the width between the two maxillary bones in the upper jaw, and head width was the width between eyes. BL, BW, MW, and HW were compared between the four groups.

### Water drinking observation

Larvae of SW-*Oj* and FW-*Oj* at 21 dph were immersed in respective media containing 1-µM fluorescein isothiocyanate (FITC)-labeled dextran (Average Molecular Weight 70,000, Sigma, 46945), which served as an inert marker for drinking. Morphological observations on drinking were made after 3 h exposure. After exposure, larvae were removed and rinsed with FW or SW for 5 min to remove excess FITC-dextran, and then anesthetized on ice. Larvae were fixed in 10% formalin overnight before observation. Individual larvae were placed in a glass-based dish, and the presence of FITC-dextran in their intestines was examined from a ventral orientation using an all-in-one fluorescence microscope (KEYENCE) equipped with a GFP filter.

### Statistical analysis

Microsoft Office 2010 and GraphPad Prism 9 were used for statistical analysis. Data are expressed as the mean ± standard error of the mean (SEM). Significant differences between seawater and freshwater groups were analyzed using one-way analysis of variance (ANOVA) followed by Tukey's Honestly Significant Difference post-hoc test. Statistical analyses of MP vertical distribution and larval body measurements were also performed using one-way ANOVA. The level of statistical significance was set at P < 0.05.

## Supplementary Information

Supplementary Figures.

Supplementary Tables.

## Data Availability

All data generated or analyzed during this study are included in this published article and its Supplementary Information files.
