# Supplementary material for: Euryhaline fish larvae ingest more microplastic particles in seawater than in freshwater
Source: Sci Rep. 2023 Mar 10;13:3560. doi: 10.1038/s41598-023-30339-y (PMC10006175; doi:10.1038/s41598-023-30339-y)
Supplement: Supplementary file 1 — Supplementary Figures. [file 41598_2023_30339_MOESM1_ESM.pdf]

Supplementary Figures

Euryhaline fish larvae ingest more microplastic particles in seawater than in freshwater

Hilda Mardiana Pratiwi[1,2], Toshiyuki Takagi[2], Suhaila Rusni[2], Koji Inoue[1,2]

[1] *Graduate School of Frontier Sciences, The University of Tokyo, Kashiwa 277-8563, Japan*

[2] *Atmosphere and Ocean Research Institute, The University of Tokyo, Kashiwa 277-8564, Japan*

*To whom correspondence should be addressed. *E-mail address*:
pratiwi.hilda.mardiana@s.nenv.k.u-tokyo.ac.jp

Page 1 – 4

Supplementary Figure S1
Supplementary Figure S2
Supplementary Figure S3

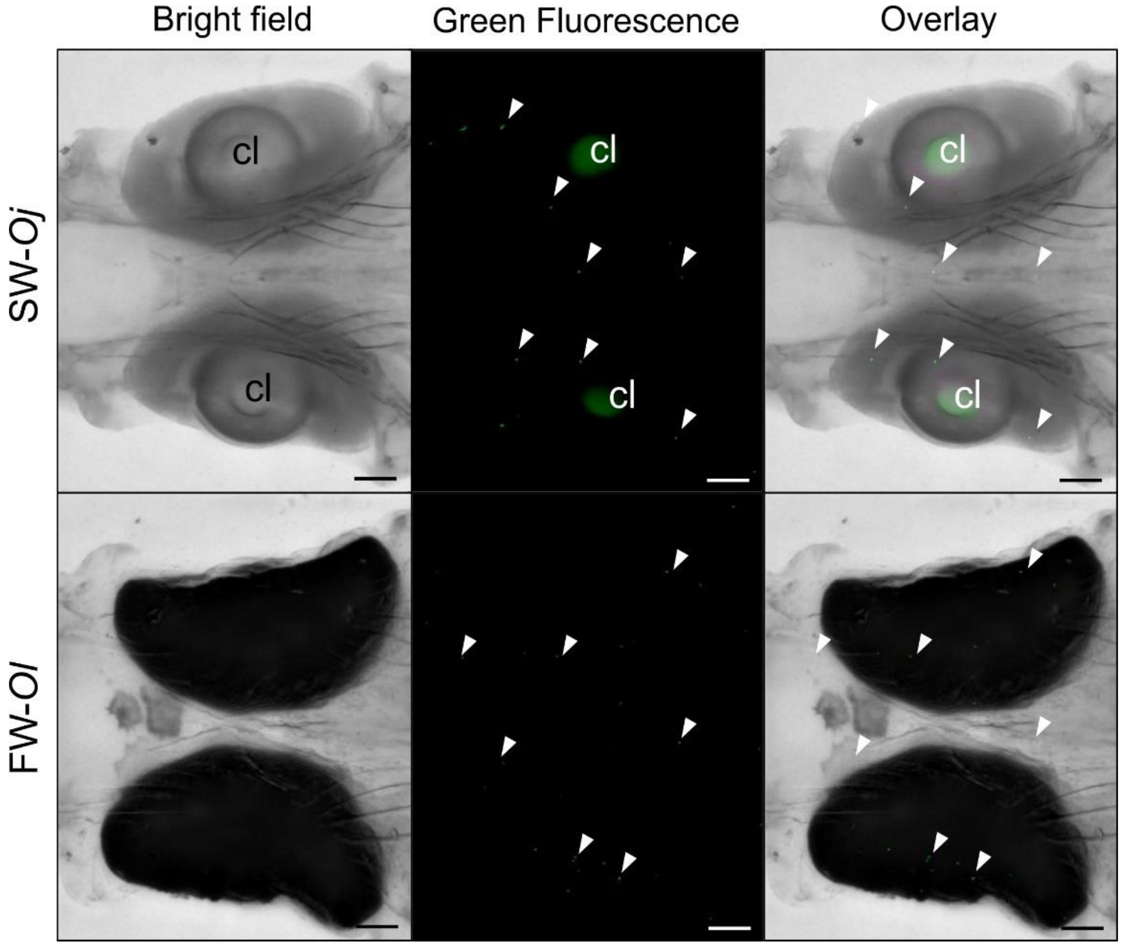

Supplementary Figure S1. Microplastic distribution in the heads of seawater (SW)-reared *Oryzias javanicus* (*Oj*) and freshwater (FW)-reared *O. latipes* (*Ol*). Images show the MP distribution in the heads of SW-*Oj* and FW-*Ol* larvae exposed to fluorescently labeled microplastics. Images were captured from the ventral side. White and black arrowheads show the green fluorescent signal from microplastic particles around eye vesicles. Autofluorescent signals from crystalline lens (cl) were detected in the SW-*Oj* sample, but no MPs were observed in this area. The scale bar indicates 100 μm.

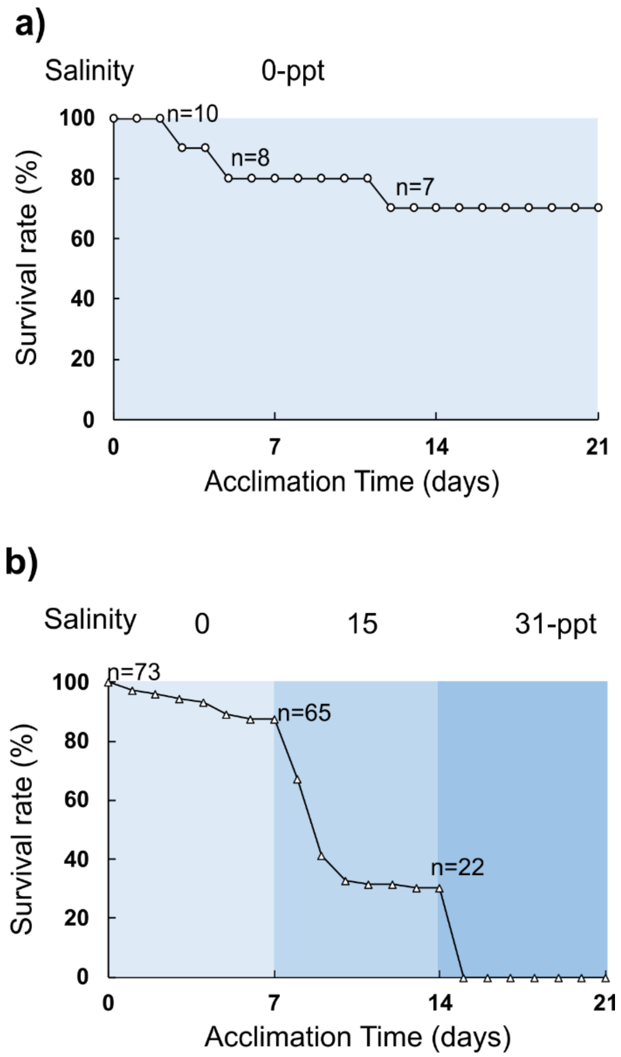

Supplementary Figure S2. Adaptability of *Oryzias latipes* larvae to different salinities.
a) 0-ppt (FW), b) 0 to 31-ppt SW. Adaptability of *O. latipes* larvae in different salinities were observed in this study. Survival rates of larvae were calculated from the percentage of dead and surviving larvae at each day during acclimation periods. Colors indicate different salinities.

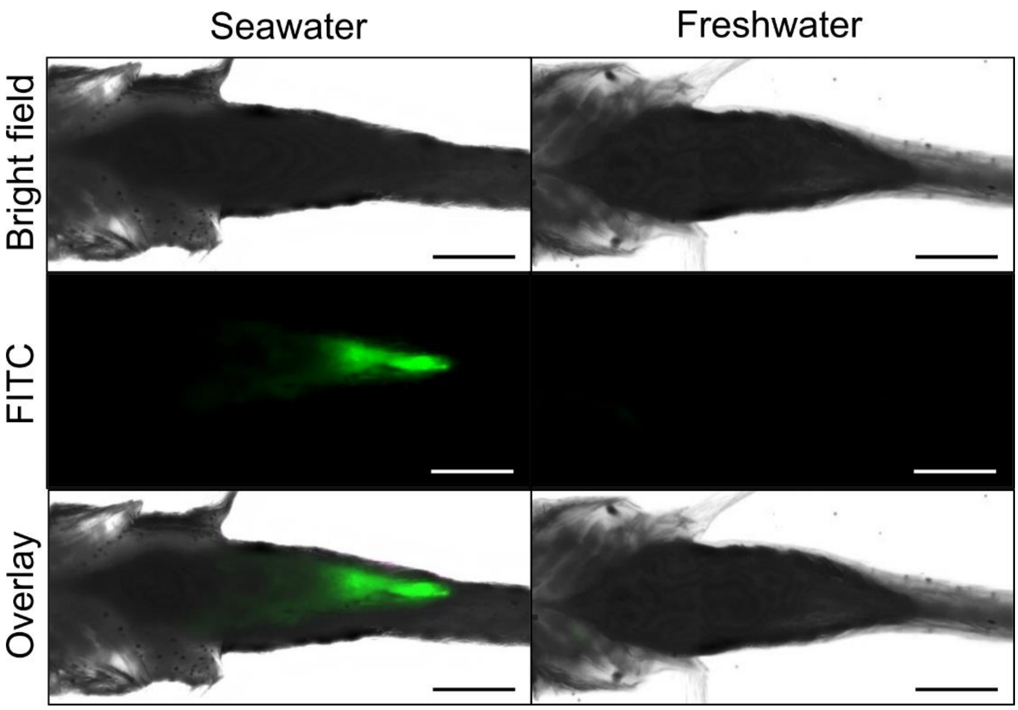

Supplementary Figure S3. Water drinking observation on 21-dph larvae of *Oryzias javanicus* reared in seawater and freshwater.

Images show larvae immersed in FITC-dextran at 1 μM for 3 h. The scale bar indicates 0.5 mm.
