# Supplementary material for: Euryhaline fish larvae ingest more microplastic particles in seawater than in freshwater
Source: Sci Rep. 2023 Mar 10;13:3560. doi: 10.1038/s41598-023-30339-y (PMC10006175; doi:10.1038/s41598-023-30339-y)
Supplement: Supplementary file 2 — Supplementary Tables. [file 41598_2023_30339_MOESM2_ESM.pdf]

Supplementary Table

Euryhaline fish larvae ingest more microplastic particles in seawater than in freshwater

Hilda Mardiana Pratiwi[1,2], Toshiyuki Takagi[2], Suhaila Rusni[2], Koji Inoue[1,2]

[1] *Graduate School of Frontier Sciences, The University of Tokyo, Kashiwa 277-8563, Japan*

[2] *Atmosphere and Ocean Research Institute, The University of Tokyo, Kashiwa 277-8564, Japan*

*To whom correspondence should be addressed. *E-mail address*:
pratiwi.hilda.mardiana@s.nenv.k.u-tokyo.ac.jp

Page 1 – 2

Supplementary Table S1

Supplementary Table S1. Water conditions before and during microplastic exposure.

Exposure Group		Temperature (°C)	Salinity (ppt)	Density (kg/m^3)
**Seawater-reared *Oryzias javanicus***	Before Exposure	26.1	31	1020.05
	During Exposure* (Day 1)	26.1	31	1020.05
	During Exposure* (Day 2)	25.9	31	1020.10
	During Exposure* (Day 3)	25.9	31	1020.11
	During Exposure* (Day 4)	26.1	31	1020.05
	During Exposure* (Day 5)	26.0	31	1020.06
	During Exposure* (Day 6)	26.0	31	1020.08
**Freshwater-reared *Oryzias javanicus***	Before Exposure	26.0	0	996.80
	During Exposure* (Day 1)	26.0	0	996.80
	During Exposure* (Day 2)	26.1	0	996.77
	During Exposure* (Day 3)	26.0	0	996.80
	During Exposure* (Day 4)	26.0	0	996.79
	During Exposure* (Day 5)	25.9	0	996.83
	During Exposure* (Day 6)	25.9	0	996.81
**Seawater-reared *Oryzias latipes***	Before Exposure	26.0	20	1011.82
	During Exposure* (Day 1)	26.0	20	1011.81
	During Exposure* (Day 2)	25.9	20	1011.84
	During Exposure* (Day 3)	25.9	20	1011.85
	During Exposure* (Day 4)	25.9	20	1011.84
	During Exposure* (Day 5)	26.0	20	1011.82
	During Exposure* (Day 6)	26.0	20	1011.81
**Freshwater-reared *Oryzias latipes***	Before Exposure	25.9	0	996.81
	During Exposure* (Day 1)	26.0	0	996.79
	During Exposure* (Day 2)	26.0	0	996.79
	During Exposure* (Day 3)	26.0	0	996.79
	During Exposure* (Day 4)	25.9	0	996.81
	During Exposure* (Day 5)	26.0	0	996.80
	During Exposure* (Day 6)	26.0	0	996.80

*** Measured about 3 hours after water replacement**
